# Molecular Characterization of Cryptically Circulating Rabies Virus from Ferret Badgers, Taiwan

**DOI:** 10.3201/eid2005.131389

**Published:** 2014-05

**Authors:** Hue-Ying Chiou, Chia-Hung Hsieh, Chian-Ren Jeng, Fang-Tse Chan, Hurng-Yi Wang, Victor Fei Pang

**Affiliations:** National Taiwan University, Taipei, Taiwan, Republic of China (H.-Y. Chiou, C.-H. Hsieh, C.-R. Jeng, H.-Y. Wang, V.F. Pang);; Council of Agriculture, Executive Yuan, Nantou County, Taiwan, Republic of China (F.-T. Chan)

**Keywords:** Rabies, *Melogale moschata subaurantiaca*, phylogeography, origin, Taiwan, ferret badger, viruses

## Abstract

After the last reported cases of rabies in a human in 1959 and a nonhuman animal in 1961, Taiwan was considered free from rabies. However, during 2012–2013, an outbreak occurred among ferret badgers in Taiwan. To examine the origin of this virus strain, we sequenced 3 complete genomes and acquired multiple rabies virus (RABV) nucleoprotein and glycoprotein sequences. Phylogeographic analyses demonstrated that the RABV affecting the Taiwan ferret badgers (RABV-TWFB) is a distinct lineage within the group of lineages from Asia and that it has been differentiated from its closest lineages, China I (including isolates from Chinese ferret badgers) and the Philippines, 158–210 years ago. The most recent common ancestor of RABV-TWFB originated 91–113 years ago. Our findings indicate that RABV could be cryptically circulating in the environment. An understanding of the underlying mechanism might shed light on the complex interaction between RABV and its host.

## Abstract 2

The virus has been circulating in Taiwan for about 100 years.

Rabies is possibly one of the oldest zoonotic diseases. It is caused by the rabies virus (RABV), a neurotropic virus in the family *Rhabdoviridae*, genus *Lyssavirus*. Except for a small number of countries and regions, particularly islands, RABV is found worldwide. The virus infects nearly all warm-blooded animals and causes severe neurologic signs, which almost invariably lead to death (*1*). It was estimated that worldwide in 2010, the disease caused >60,000 human deaths, primarily in Africa and Asia (*2*). Although dogs are considered the principal host of RABV in developing countries, the virus is also dispersed among many species of wild carnivora and chiroptera, especially in those countries of Europe and North America that have well-established vaccination programs (*3*). Mustelids, including various species of the genera *Melogale*, *Meles*, and *Mellivora* of the weasel family Mustelidae, can carry RABV (*4*–*6*). In southeastern China, Chinese ferret badgers (CNFB; *Melogale moschata moschata*) have been associated with human rabies for many years and are considered to be a primary host in this region (*7*–*9*).

After what were considered to be the last reported cases of rabies in a human and a nonhuman animal in 1959 and 1961, respectively, Taiwan was rabies free for >50 years until the 2012–2013 outbreak of ferret badger–associated rabies. During May 2012–January 2013, through a government-supported program of routine disease surveillance of free-range dead wild animals that had been killed by vehicles or were receiving treatment for injuries and/or illness at the wildlife first aid station, 3 dead Taiwan ferret badgers (TWFB; *M. moschata subaurantiaca*) were submitted to the School of Veterinary Medicine, National Taiwan University, for further examination. 

Pathologic examination revealed nonsuppurative meningoencephalomyelitis with formation of eosinophilic intracytoplasmic inclusion bodies in all 3 animals; reverse transcription PCR and immunohistochemical staining excluded the possibility of infection with the canine distemper virus. However, the results of fluorescence antibody testing, immunohistochemical staining, and reverse transcription PCR, followed by sequencing for RABV, were positive (H.-Y. Chiou, unpub. data). After the rabies diagnoses for the initial 3 ferret badgers were confirmed, by the end of August of 2013, rabies had been diagnosed by fluorescence antibody testing for an additional 105 dead or ill and euthanized ferret badgers and 1 shrew.

Our objective in this study was to clarify whether the current outbreak of the TWFB–associated rabies is an emerging, a reemerging, or a cryptically circulating disease. We investigated the possible origin of this outbreak and its relations with CNFB-associated rabies in mainland China via genomic organization and characterization and analysis of genetic diversity and phylogeographic origin of RABV-TWFB. In addition, we propose a mechanism that might be contributing to the limited host range of RABV-TWFB.

## Materials and Methods

### Animals and Specimen Collection

**Figure 1:**
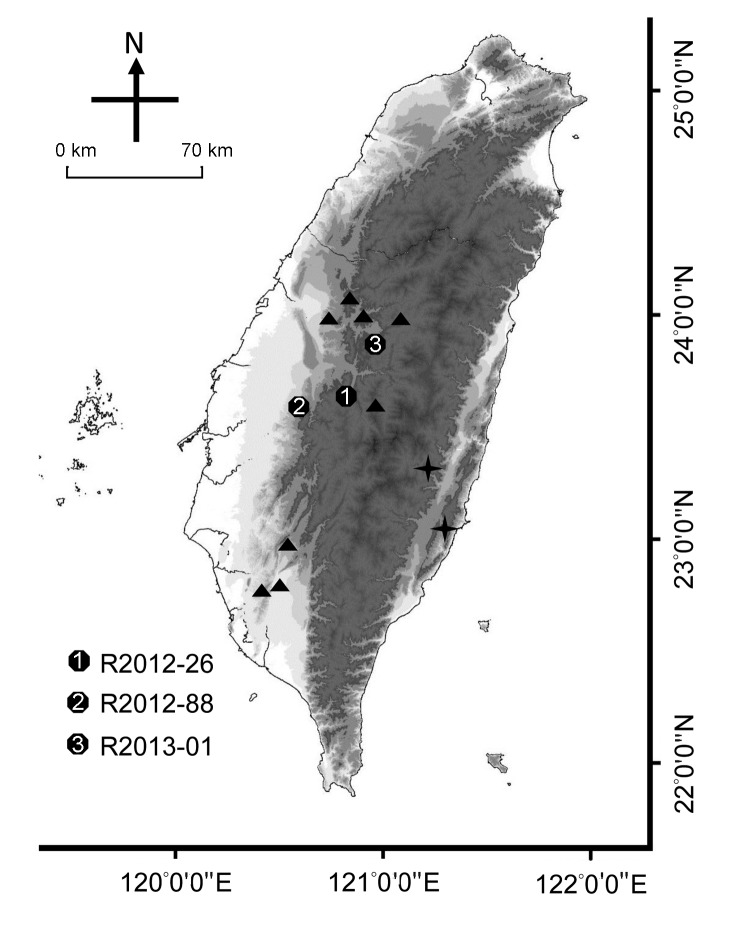
Collection sites of rabies-positive Taiwan ferret badgers (TWFB), Taiwan. Solid circles marked with 1–3 represent the collection sites of the first 3 rabies-positive animals. Triangles represent the collection sites of other rabies virus (RABV) sequences included in this study. Crosses represent the most diverged lineages of rabies virus from Taiwan ferret badgers (TWFB, TW1614, and TW1955), shown in Figure 5, panel B, Appendix, and the easternmost cross represents the isolate from a shrew, TW1955.

During May 2012–January 2013, three ill TWFB were collected from different regions of central Taiwan (Figure 1). One was in the Xitou nature education area at Lugu Township, Nantou County (R2012–26); one was in Gukeng Township, Yunlin County (R2012–88); and one was in Yuchih Township, Nantou County (R2013–01). These 3 TWFB, respectively, showed the following clinical signs: emaciation, coma, paddling, loss of pain response, reduced body temperature, and a 2-cm skin wound on the chin; extreme weakness and inability to move; and signs of weakness and respiratory signs, including labored breathing and increased breath sounds with hypersalivation and exudation of foamy fluid from the mouth and nose. Initial supportive treatment was provided at the wildlife first aid station, but the ferret badgers died within 1–3 days, and their carcasses were submitted to the School of Veterinary Medicine, National Taiwan University, for routine disease surveillance. Full necropsy was performed, during which half of the left cerebral hemisphere was collected from each animal and stored at −80°C for subsequent nucleic acid extraction. Representative tissue samples were taken from all major organs and fixed in 10% neutral buffered formalin for histopathologic examination.

### Sample Preparation and Genome Sequencing

Approximately 25 mg of brain specimen from each animal was homogenized, and 1 mL of TRIzol reagent (Invitrogen, Carlsbad, CA, USA) was added. Total RNA was extracted by using an RNeasy Mini Kit (QIAGEN, Valencia, CA, USA), and cDNA was synthesized by using a Transcriptor First Strand cDNA Synthesis Kit (Roche Diagnostics, Indianapolis, IN, USA) according to the manufacturer’s instructions. To amplify the whole genome, we used 19 pairs of primers (Table 1), including the forward primer for the 5′ end and the reverse primer for the 3′ end designed to be complementary to the respective ends of the genome, as described (*10*).

**Table 1:** Primers used for the amplification and sequencing of rabies virus genome*

Primer	Sequence, 5′→3′	Amplicon size, nt	Position, nt
3′F	GTACCTAGACGCTTAACAAC	499	1–499
3′R	AAGACCGACTAAGGACGCAT		
NF	ATGTAACACCTCTACAATGG	1,533	55–1587
NR	CAGTCTCYTCNGCCATCT		
PF	GAACCAYCCCAAAYATGAG	1,001	1500–2500
PR	TTCATTTTRTYAGTGGTGTTGC		
MF	AAAAACRGGCAACACCACT	641	2479–3119
MR	TCCTCYAGAGGTAWACAAGTG		
G1F	TGGTGTATAACATGRAYTC	1,097	3000–4096
G1R	ACCCATGTYCCRTCATAAG		
G2F	TGGATTTGTGGATKAAAGAGGC	1,542	3995–5536
G2R	GAGTTNAGRTTGTARTCAGAG		
L1F	TGGRGAGGTYTATGATGACCC	726	5430–6155
L1R	CAGCATNAGTGTRTAGTTTCTGTC		
L2F	GGTCGATTATGATAAKGCATTTGG	704	5885–6588
L2R	TTGACAGACCCTTTCGATAATC		
L3F	GGATCAATTCGACAACATACATG	550	6473–7024
L3R	AAGTCTTCATCHGGCARTCCTCC		
L4F	AGACTAGCTTCHTGGYTGTCAG	708	6882–7589
L4R	TACTTTGGTTCTTGTGTTCCTG		
L5F	AGTGTTTGGATTGAAGAGAGTGTT	662	7337–7998
L5R	GAAAGACTGCCTGCACTGACAT		
L6F	AATAGTCAACCTCGCCAATATAATG	767	7897–8645
L6R	GGATCTCTGAGTTGTAGAAGGATTC		
L7F	CCGAGTCAATCATTGGATTGATAC	621	8517–9137
L7R	GAATACCCTCCTTCGCTGTATCTG		
L8F	GAGAAGGTCACCAATGTTGATG	1,045	8958–10002
L8R	AGATCCAYARCCAGTCATTCTC		
L9F	ACATAATGCTCAGAGAACCGT	503	9820–10322
L9R	CCATTCTGAACATCCTACCTT		
L10F	TGTTCAGAATGGGTCTGCTCT	509	10302–10811
L10R	TGCATCGCAAATAATGAGGT		
L11F	ATTATTTGCGATGCAGAAGT	524	10797–11320
L11R	ATGATAGCCACTTTAGACAGAGT		
L12F	GTTACAGAGGGGAACTCTGTCT	386	11285–11670
L12R	TCTTCACTATCTTGTAAATCAACCT		
5′F	TGGATCAGGTTGATTTACAAGATAGT	293	11640–11932
5′R	ACGCTTAACAAATAAACAACAAAAAT		

*Primers were from Lei et al. (*10*).

### Sequence Analyses and Phylogenetic Reconstruction

Sequences were assembled by using the Seqman program (Lasergene 8, Madison, WI, USA) (GenBank accession nos. KF620487–KF620489) and then aligned by using the ClustalW program (*11*). The genetic distance was estimated by using the Kimura 2-parameter substitution model implemented in MEGA version 5.0 (*12*). The nucleotide diversity within populations was calculated by using DnaSP version 5.0 (*13*). To test for the deviation of neutral expectation, we conducted the Tajima D (*14*) and the Fu and Li D* (*15*) tests implemented in DnaSP. Significance was assessed by 10^4^ coalescent simulations (*13*).

To investigate the phylogenetic position of RABV-TWFB isolates, we included 24 complete RABV genomes representing the 3 major phylogenetic groups (*16*). For global phylogeny of RABV, we analyzed 218 full-length (1,335-nt) sequences of the nucleoprotein (N) gene, including 11 sequences from Taiwan. We also analyzed 125 full-length (1,575-nt) sequences of the glycoprotein (G) gene, including 13 sequences from Taiwan (*17*). For each gene, phylogenetic trees were inferred by using maximum-likelihood and Bayesian inference methods.

The maximum-likelihood analysis was conducted by using PhyML 3.0 online (*18*); the starting tree was derived from the neighbor-joining method, and the nearest neighbor interchange topology search option was used. The nucleotide substitution model for phylogenetic reconstruction was determined by using the Akaike information criterion implemented in jModeltest 0.1.1 (*19*). The method of Bayesian inference was performed by using MrBayes version 3.1.2 (*20*). Analyses were initiated with random starting trees, and Metropolis-coupled Markov chain Monte Carlo (MCMC) analyses were run for 1 × 106 generations and sampled every 100 generations. The steady state of the log-likelihood was reached at ≈20,000 generations. Subsequently, the first 201 trees were excluded and the remaining 9,800 trees were retained to compute a 50% majority-rule consensus tree.

### Divergence Dating

The divergence time between different viral lineages and the time to the most recent common ancestor (TMRCA) of virus isolates were estimated by using an established Bayesian MCMC approach implemented in BEAST version 1.7 (*21*). The analysis was performed by using the general time-reversible model of nucleotide substitution assuming an uncorrelated lognormal molecular clock (*22*). We linked substitution rates for the first and second codon positions and allowed independent rates in the third codon position. The molecular clocks were 2.3 × 10–4 (range 1.1–3.6 × 10–4) and 3.9 × 10–4 (1.2–6.5 × 10–4) substitutions/site/year for N and G genes, respectively (*17*). A slightly faster clock, 4.3 × 10–4 (3.1–5.6 × 10–4) substitutions/site/year for N gene (*23*), was also used in a separate analysis.

Because a previous study revealed that the population dynamics of RABV supported a model of constant population size through time (*17*), we restricted our analysis to this demographic model. For each analysis, we performed 2 independent runs with 2 × 107 MCMC steps, of which the first 10% were discarded as burn-in. To confirm that both were sampling the same distribution, we compared and then combined the results. Log files were checked by using Tracer (http://beast.bio.ed.ac.uk/Tracer), and the effective sample size for each parameter was >300, which is adequate according to the authors of BEAST software.

## Results

### Genomic Organization and Characterization of RABV-TWFB

**Figure 2:**
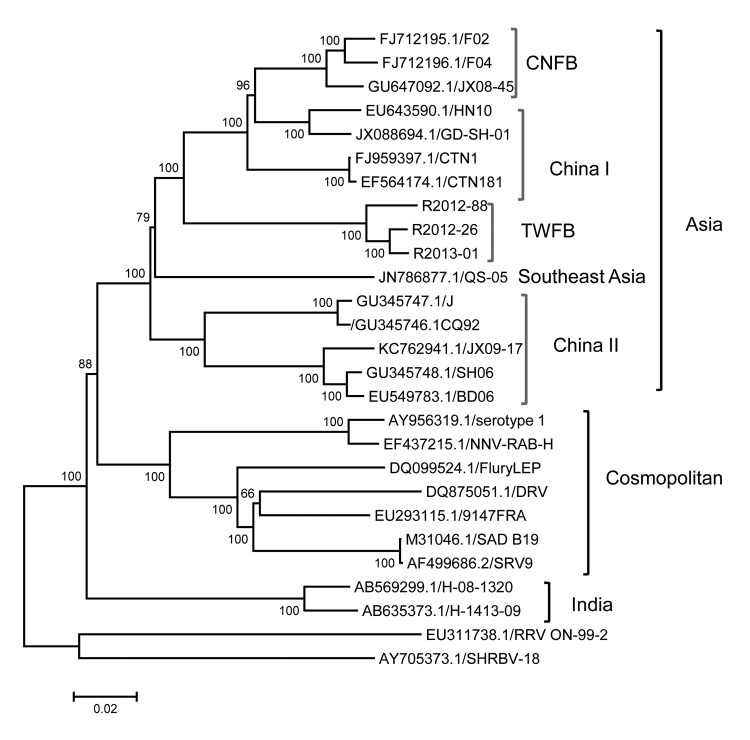
Phylogenetic relationships of 27 rabies virus (RABV) genomes constructed by maximum-likelihood method. Numbers close to the nodes were from 1,000 bootstrap replications. The tree was rooted with RABV from bats and raccoons. Three major groups, Asia, Cosmopolitan, and India, are strongly supported, as indicated (*17*). There are 4 major lineages within the group from Asia, including previously recognized China I, China II (*16*), Southeast Asia, and RABV from Taiwan ferret badgers (TWFB). RABVs derived from Chinese ferret badgers (CNFB) are clustered with China I, indicating that RABVs of TWFB and CNFB are of independent origin. Scale bar indicates nucleotide substitutions per site.

Similar to previous analyses (*16*,*17*), our phylogenetic analysis that used whole RABV genomes revealed 3 major groups with high bootstrap support (Figure 2). Although the 3 RABV-TWFB isolates are clustered within the Asia group, composed of 3 distinct lineages, (China I [including CNFB], China II [*16*], and Southeast Asia), they do not appear close to any of the 3 lineages. More noteworthy, the 3 isolates of RABV-TWFB are not close to those of RABV-CNFB, indicating that they may have originated independently.

The genome of RABV-TWFB is 11,923 nt long and encodes 5 proteins. The nucleotide lengths of different genomic regions are within the range of variations in different Asia lineages (Table 2), except the matrix protein (M)-G intergenic region, which is 1 nt longer than in the rest of the lineages from Asia (212 vs. 211). Within the group of Asia lineages, the most conserved protein is M, followed by N, the virion-associated RNA polymerase (L), and G; the least conserved is phosphoprotein (P) (Table 3). Among the RABV groups, however, N becomes the most conserved followed by L, M, G, and P. The RABV-TWFB is closest to China I lineage in the N, P, and L gene regions, but it is closest to RABV-CNFB in the M and G gene regions.

**Table 2:** Genomic organization and nucleotide lengths of terminal and intergenic regions and viral genes of rabies virus from Taiwan and other lineages from Asia*

Isolate	3′-UTR	Gene, nucleotide length									5′-UTR	Genome size
		N	N–P	P	P–M	M	M–G	G	G–L	L		
TWFB	70	1,353	91	894	86	609	212	1,575	519	6,384	130	11,923
CNFB	70	1,353	91	894	86	609	211	1,575	519	6,384	130–131	11,922–11,923
China I†	70	1,353	90–91	894	87–88	609	211	1,575	519	6,384	130	11,923
China II†	70	1,353	91	894	87	609	211	1,575	518–519	6,384	131	11,923–11,924

*UTR, untranslated region; TWFB, Taiwan ferret badgers; CNFB, Chinese ferret badgers.  †The definitions of China I and II are based on He et al. (16). China I does not include CNFB.

**Table 3:** Genetic distances between Taiwan isolates and other rabies virus lineages or groups in different genomic regions*

Rabies virus group†	Gene					
	N	P	M	G	L	Genome
Asia						
CNFB	0.115	0.172	0.105	0.129	0.130	0.134
China I‡	0.104	0.157	0.107	0.133	0.123	0.127
China II	0.130	0.186	0.132	0.172	0.142	0.152
Southeast Asia	0.140	0.189	0.125	0.169	0.147	0.155
Cosmopolitan	0.161	0.216	0.173	0.209	0.177	0.192
India	0.152	0.229	0.183	0.211	0.175	0.191
Outgroup	0.200	0.284	0.212	0.237	0.209	0.232

*CNFB, Chinese ferret badgers; outgroup, rabies virus derived from bat and raccoon. †Groups of rabies virus are based on the work of Bourhy et al. (*17*). ‡In this analysis, China I does not include CNFB.

**Figure 3:**
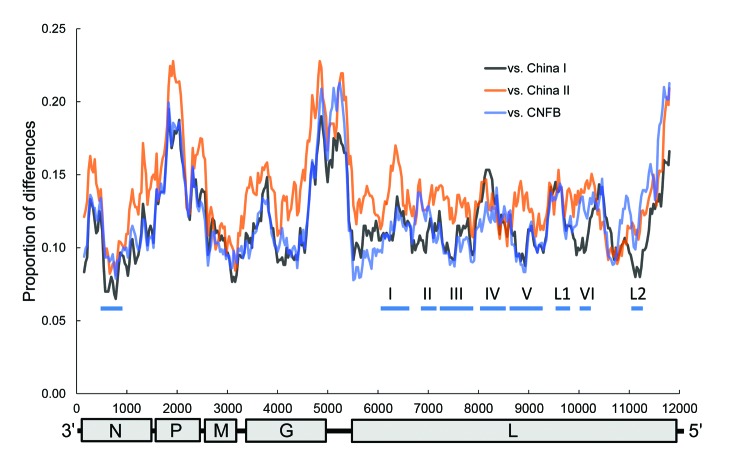
Sliding window analysis of rabies virus (RABV) genetic variations between Taiwan ferret badgers and China I, China II, and Chinese ferret badgers (CNFB). The genomic organization of RABV is shown at the bottom with nucleotide positions on the x-axis. The thick horizontal lines indicate conserved regions across lyssaviruses. N, nucleoprotein; P, phosphoprotein; M, matrix protein; G, glycoprotein; L, virion-associated RNA polymerase.

The genetic variations across the whole genome among different lineages can be viewed in a sliding window analysis (Figure 3). Within N, there seems to be a conserved central domain previously identified in RABV at residues 182–328 (*24*), which is also conserved in RABV-TWFB. The P, the last quarter of G and G–L intergenic regions, and the last part of L are more variable among different lineages of RABV than is the rest of the genome. The conserved M is functional in viral assembly and budding (*25*); is involved in the regulation of transcription and replication of viral RNA (*26*); and has been reported to induce apoptosis (*27*), suggesting its role in host-cell interplay. The involvement of M in multiple interactions explains its conservation among lineages. G is responsible for cell attachment and fusion and is the main viral protein responsible for the induction of neutralization antibodies and cell-mediated immune responses. The region between aa 189 and aa 214, proposed to be needed for G binding to the nicotinic acetylcholine receptor (*28*), is relatively conserved in the dataset. Nevertheless, 3 substitutions (N194Y, R196K, and G203E) are found exclusively in RABV-TWFB G. In L, Poch et al. (*29*) recognized 6 conserved blocks, including B1 (233–424), B2 (504–608), B3 (609–832), B4 (890–1061), B5 (1091–1326), and B6 (1674–1749) (*29*). In addition, 2 regions, L1 (1418–1515) and L2 (1884–1961), are also conserved across lyssaviruses (*30*). In RABV-TWFB, the B4 and L1 regions in L are variable, and the rest of the blocks are conserved (Figure 3).

### Genetic Diversity and Phylogeographic Origin of RABV-TWFB

**Figure 4:**
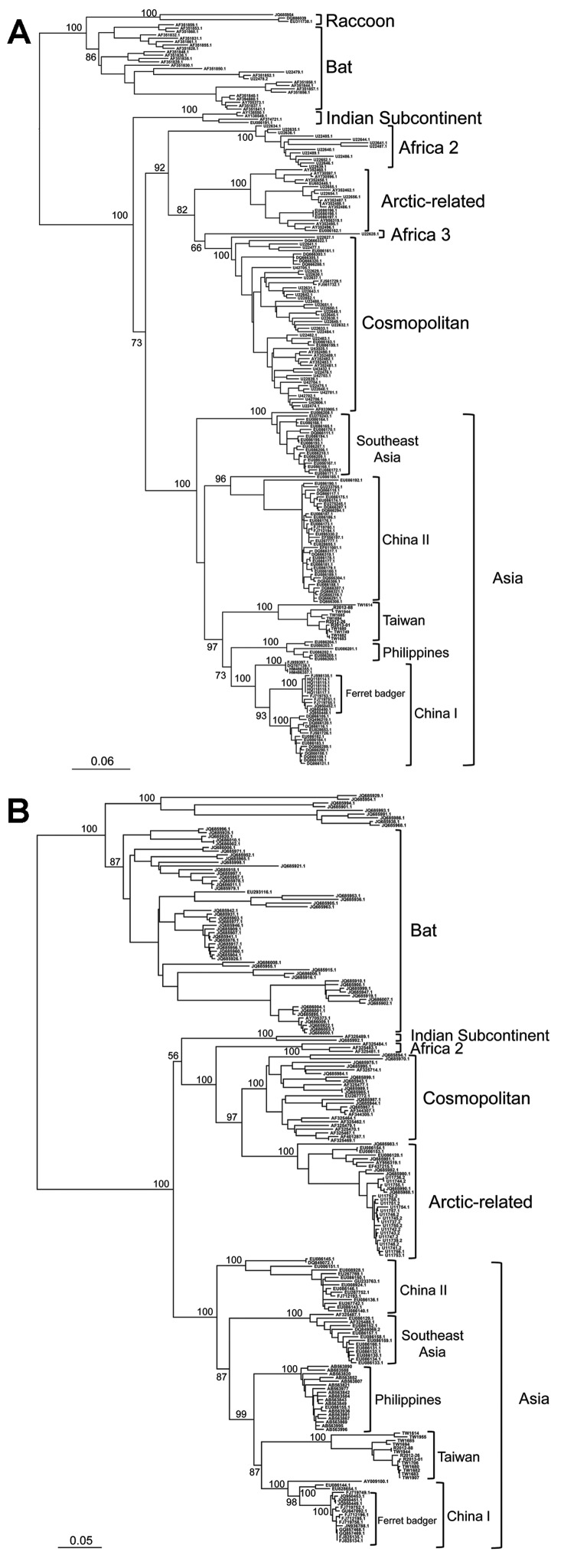
Phylogenetic relationships of major rabies virus groups based on (A) nucleoprotein and (B) glycoprotein gene sequences. The trees were constructed by the maximum-likelihood method based on the general time-reversible nucleotide substitution model. Numbers close to the node are from 100 bootstrap replications. The definition of major clades was based on the work of He et al. (*16*) and Bourhy et al. (*17*). Scale bar indicates nucleotide substitutions per site.

The data shown in Figure 2 indicate that RABV-TWFB is a distinct lineage within the Asia group of viruses. To further explore the detailed origin of RABV-TWFB, we included the representative N and G sequences of RABV from human and various animal species for analysis (*17*,*31*). Because maximum-likelihood and Bayesian inference methods yielded similar topologies, we report only the results derived from the former. Both N and G gene trees support the conclusion that RABV-TWFB is a distinct lineage within the Asia group, clustered with the China I lineage, including RABV-CNFB, and sequences from the Philippines (Figure 4, Appendix).

**Figure 5:**
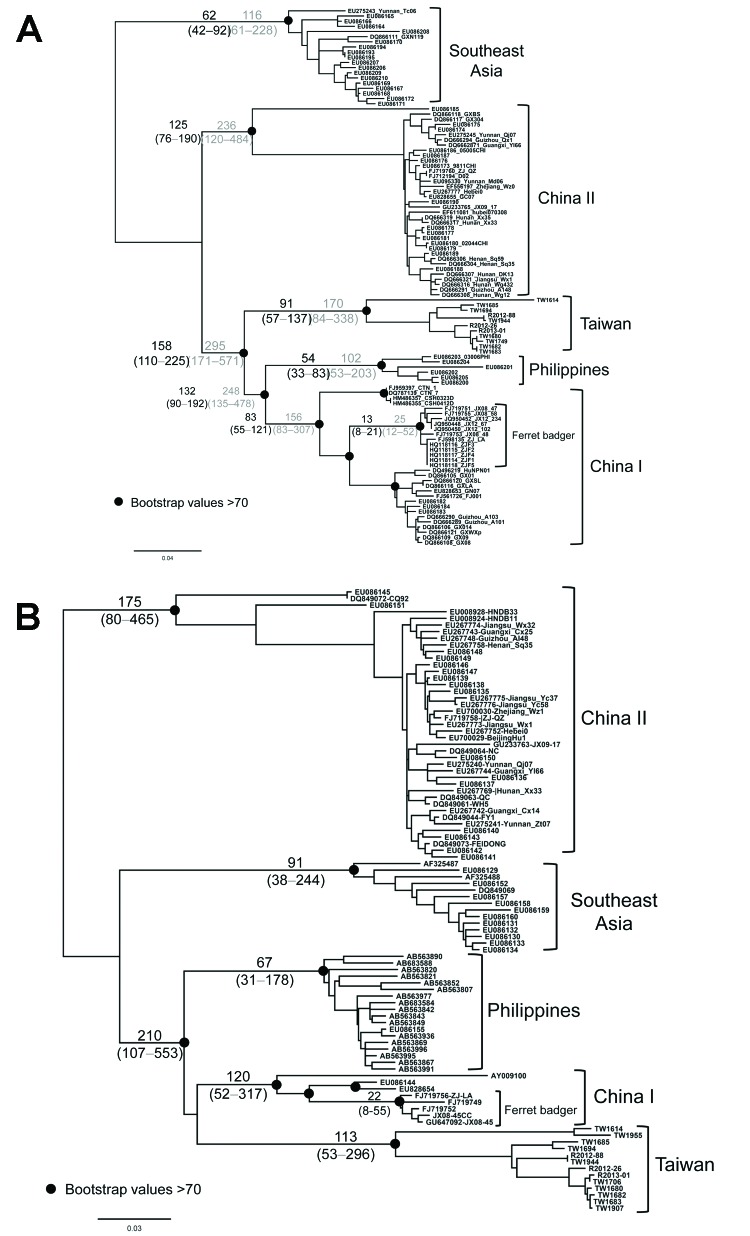
Maximum-likelihood trees of rabies virus based on (A) nucleoprotein and (B) glycoprotein gene sequences. Numbers on the branches are estimated divergences (above) and their 95% highest posterior density (below). The divergence time between different viral lineages and time to the most recent common ancestor of virus isolates were estimated by using an established Bayesian Markov chain Monte Carlo approach implemented in BEAST version 1.7 (*21*). The substitution rate was assumed to be 4.3 × 10–4 (range 3.1–6.6 × 10–4) or 2.3 × 10–4 (range 1.1–3.6 × 10–4)/sites/year (hatched numbers) for nucleoprotein (N) and 3.9 × 10–4 (1.2–6.5 × 10–4)/sites/year for glycoprotein (G) genes (*17**,**23*). The analysis was performed by using the general time-reversible model of nucleotide substitution, assuming an uncorrelated lognormal molecular clock (*22*). TW1955 is the only isolate from a shrew that is very close to TW1614 collected from the same area, suggesting that TW1955 was a spillover from a ferret badger (see also Figure 1). Scale bar indicates nucleotide substitutions per site.

Divergence time was estimated by using a Bayesian coalescent approach. In this analysis, we included only sequences of the Asia group. On the basis of the molecular clock of 4.3 × 10–4/site/year for N gene (*23*), the substitution rate at the third codon position is 1.1 × 10–3/site/year, and RABV-TWFB was separated from China I and the Philippines isolates 158 years ago with 95% highest posterior density (HPD) ranging from 110 to 225 years (Figure 5, panel A, Appendix). The divergence between China I and the Philippines isolates occurred 132 (95% HPD, 90–192) years ago, which is similar to previous estimations (*23*,*32*). The TMRCA of isolates from Taiwan was 91 years (95% HPD, 57–137). A similar timescale, with overlapping 95% HPD, was derived by using the molecular clock of 2.3 × 10–4/site/year for N gene (*17*) (Figure 5, panel A, hatched numbers, Appendix). The mean substitution rate for G gene sequences was 3.5 × 10–4/site/year (7.8 × 10–4 for the third codon position), and the divergence of RABV-TWFB, China I, and the Philippines isolates was initiated 210 (107–553) years ago, and the TMRCA of isolates from Taiwan was 113 (53–296) years (Figure 5, panel B, Appendix). It is notable that the TMRCA of RABV-TWFB was more ancient than that of several distinct lineages in Figure 5, Appendix. For example, the TMRCA was 62–116 years for the Southeast Asia lineage and 54–102 years for RABV of the Philippines. The origin of RABV-CNFB was relatively recent; TMRCA was 13–25 years.

The nucleotide diversities of RABV-TWFB are 3.14% for the N and 4.21% for the G genes (Table 4), which are almost 5 times higher than those of RABV-CNFB, which are 0.67% for the N and 0.87% for G genes. For comparison purposes, the 65 N and 232 G gene sequences of RABV isolates from the Philippines were also included for analysis (*33*). The nucleotide diversities are 2.00% for the N gene and 2.57% for the G gene. The results of both the Tajima D and the Fu and Li D* tests are not significant for RABV-TWFB, indicating that the viral population is under neutral equilibrium, which in turn suggests that RABV was not recently introduced to TWFB. In contrast, the results of the Tajima D and the Fu and Li D* tests are significantly negative for the sequences of RABV-CNFB and sequences from the Philippines isolates, which are caused by an excess of low-frequency mutation or by differentiation among populations.

**Table 4:** Nucleotide diversity of rabies virus from TWFB, CNFB, and the Philippines*

Virus, gene	Length, nt	Sample size, no.	Π, %	Tajima D	Fu and Li D*
TWFB					
N	1,335	11	3.14	−0.886	0.735
G	1,575	12	4.21	−0.264	0.740
CNFB					
N	1,350	12	0.67	−1.294†	−1.918†
G	1,575	14	0.87	−1.168†	−1.336†
Philippines					
N	1,124	65	2.00	−1.380†	−1.759†
G	1,575	232	2.57	−1.676†	−3.80‡

*TWFB, Taiwan ferret badgers; CNFB, Chinese ferret badgers. †p<0.05. ‡p<0.01.

## Discussion

### The Ancient Origin of RABV-TWFB

We sequenced and characterized a RABV strain, RABV-TWFB, recently isolated from ferret badgers in Taiwan. Our data showed that RABV-TWFB is clustered with sequences from the Philippines, China I, and RABV-CNFB. This relationship is strongly supported on the basis of multiple sequences of the N and G genes and of the complete genome (Figures 2 and 4, Appendix). Of ferret badger isolates, RABV-TWFB and RABV-CNFB come from phylogenetically distinct lineages, indicating that multiple RABV colonization events in this species probably occurred. A major question addressed in this study is whether RABV was recently introduced into the population of TWFB or perpetuated in TWFB without revealing its presence pathogenically after it was first introduced in the ancient past. Our divergence dating showed that the RABV has been circulating in TWFB for ≈100 years.

Our divergence and TMRCA estimations have a few potential sources of error. First, the RABV isolates from Taiwan might have originated from several introduction events, including the probability that multiple viral lineages occurred in the recent past and that the inflated TMRCA resulted from the combination of different, highly differentiated virus strains. Nevertheless, all isolates from Taiwan formed a monophyletic lineage distinct from other virus isolates. Unless several undetected virus strains were circulating around Taiwan, which is highly unlikely, the phylogenetic analyses support the existence of only 1 origin of RABV-TWFB. 

Second, the ancient estimates could have resulted from the application of an inadequate molecular clock. However, the nucleotide substitution (mutation) rates of 2.3–4.3 × 10–4 and 3.9 × 10–4/site/year, for the N and G genes, respectively, used in the study reported here are in agreement with findings of other studies of lyssavirus evolution (*17*,*23*,*32*,*34*,*35*). In a study of RABV in bats, Streicker et al. (*34*) found that the nucleotide substitution rates in the third codon position, which are predominately silent (synonymous) substitutions, among viral lineages in different bat species spanned 8.3 × 10–5-2.1 × 10–3/site/year. Our estimations of mutations of 1.1 × 10–3 and 7.8 × 10–4/sites/year for the third codon position of the N and G genes, respectively, are actually close to the upper boundary of their estimations. Therefore, our results should be conservative. 

Third, RABV-TWFB exhibits high nucleotide diversity in the N and G genes. Notably, 232 G gene sequences collected from a large area of the Philippines showed nucleotide diversity that was two thirds that of RABV-TWFB. Taken together, all current genetic evidence supports the hypothesis of the ancient origin of RABV-TWFB. In addition, RABV-TWFB has been maintained in a large population for a long time.

Last, our recent retrospective study that used the archived formalin-fixed and paraffin-embedded brain tissues of ferret badgers, kindly provided by various institutes, demonstrated that the current earliest TWFB-associated RABV infection could be traced back to 2004 (H.-Y. Chiou, unpub. data), representing the oldest specimens that we have so far. That finding is consistent with the notion of a long history of RABV-TWFB in Taiwan.

### Mutations in the G Gene of RABV-TWFB

The ancient history of RABV-TWFB raises 2 issues. First, because for the past 50 years Taiwan was believed to have been free from rabies, learning that the virus must have been cryptically circulating in the environment for such a long time is surprising. Because previous rabies surveillance was mainly focused on dogs and bats (www.baphiq.gov.tw), cases in remote areas might have gone unnoticed. However, Taiwan is an island with a high population density; 23 million persons live in an area of 36,188 km2, and for rabies cases to have gone unnoticed for >50 years would be very unusual. Second, according to a recent survey about wildlife, the ferret badger population has been increasing in the past 5 years (L.-K. Lin, pers. comm.). Therefore, despite the ancient history of the ferret badger’s association with RABV, the fact that its population is seemingly unaffected by infection with RABV is perplexing.

Except for 1 isolate from a shrew, all RABV isolates in the recent rabies outbreak in Taiwan have come from ferret badgers. The close relationship between the shrew RABV and ferret badger RABV collected from the same area suggests that the former probably resulted from spillover from the latter (Figure 5, panel B, Appendix). According to the most recent rabies surveillance data, the ferret badger is probably the only source of RABV in the current outbreak in Taiwan. Speculation that this RABV strain has adapted to and has been circulating in TWFB for a long time is reasonable. Its ability to transmit across species (e.g., ferret badger to shrew) is, thus, worthy of further investigation.

Among the multiple substitutions in RABV-TWFB genome that distinguish it from other virus strains, several substitutions in G (i.e., N194Y, R196K, and G203E) might merit additional attention. It has been demonstrated that a single amino acid mutation, N194K, in the nonpathogenic RABV vaccine strain SAD B19 was solely responsible for its increased pathogenicity. The increased pathogenicity is caused by increased virus spread in vivo and faster internalization of the virus into cells (*36*), both of which are consistent with the notion that G plays a major role in RABV pathogenesis. When the amino acid N was exchanged with amino acid S at position 194 (N194S), the pathogenic phenotype was reversed (*36*,*37*). Faster internalization of the virus into cells after N194K substitution might suggest that this region has some role in host cell binding. Evidence is strong that the muscular form of the nicotinic acetylcholine receptor, the neuronal cell adhesion molecule, and the p75 neurotrophin receptor serve as receptor sites for RABV binding and/or facilitate its entry into host cells (*38*,*39*). The 3 above-mentioned substitutions in RABV-TWFB are located in the region (aa 189 and 214) proposed to be needed for the binding of the G to the nicotinic acetylcholine receptor (*28*).

In our analysis of 113 G gene sequences from dog-associated RABV, no amino acid substitution was observed at the above-mentioned sites. In a total of 120 G gene sequences from bat-associated RABV (*40*), 8 N194T (6.7%), 2 N194S (1.7%), and 36 R196K (30.0%) amino acid substitutions were revealed with no amino acid change at G203. Taken together, the 3 aa substitutions (N194Y, R196K, and G203E) found in all 13 G gene sequences of RABV-TWFB are unique and worthy of further investigation.
